# Developing a Machine Learning–Based Automated Patient Engagement Estimator for Telehealth: Algorithm Development and Validation Study

**DOI:** 10.2196/46390

**Published:** 2025-01-20

**Authors:** Pooja Guhan, Naman Awasthi, Kathryn McDonald, Kristin Bussell, Gloria Reeves, Dinesh Manocha, Aniket Bera

**Affiliations:** 1 Department of Computer Science University of Maryland College Park, MD United States; 2 Department of Psychiatry, Child and Adolescent Division University of Maryland Baltimore, MD United States; 3 School of Nursing University of Maryland Baltimore, MD United States; 4 Department of Computer Science Purdue University West Lafayett, IN United States

**Keywords:** machine learning, mental health, telehealth, engagement detection, patient engagement

## Abstract

**Background:**

Patient engagement is a critical but challenging public health priority in behavioral health care. During telehealth sessions, health care providers need to rely predominantly on verbal strategies rather than typical nonverbal cues to effectively engage patients. Hence, the typical patient engagement behaviors are now different, and health care provider training on telehealth patient engagement is unavailable or quite limited. Therefore, we explore the application of machine learning for estimating patient engagement. This can assist psychotherapists in the development of a therapeutic relationship with the patient and enhance patient engagement in the treatment of mental health conditions during tele–mental health sessions.

**Objective:**

This study aimed to examine the ability of machine learning models to estimate patient engagement levels during a tele–mental health session and understand whether the machine learning approach could support therapeutic engagement between the client and psychotherapist.

**Methods:**

We proposed a multimodal learning-based approach. We uniquely leveraged latent vectors corresponding to affective and cognitive features frequently used in psychology literature to understand a person’s level of engagement. Given the labeled data constraints that exist in health care, we explored a semisupervised learning solution. To support the development of similar technologies for telehealth, we also plan to release a dataset called Multimodal Engagement Detection in Clinical Analysis (MEDICA). This dataset includes 1229 video clips, each lasting 3 seconds. In addition, we present experiments conducted on this dataset, along with real-world tests that demonstrate the effectiveness of our method.

**Results:**

Our algorithm reports a 40% improvement in root mean square error over state-of-the-art methods for engagement estimation. In our real-world tests on 438 video clips from psychotherapy sessions with 20 patients, in comparison to prior methods, positive correlations were observed between psychotherapists’ Working Alliance Inventory scores and our mean and median engagement level estimates. This indicates the potential of the proposed model to present patient engagement estimations that align well with the engagement measures used by psychotherapists.

**Conclusions:**

Patient engagement has been identified as being important to improve therapeutic alliance. However, limited research has been conducted to measure this in a telehealth setting, where the therapist lacks conventional cues to make a confident assessment. The algorithm developed is an attempt to model person-oriented engagement modeling theories within machine learning frameworks to estimate the level of engagement of the patient accurately and reliably in telehealth. The results are encouraging and emphasize the value of combining psychology and machine learning to understand patient engagement. Further testing in the real-world setting is necessary to fully assess its usefulness in helping therapists gauge patient engagement during online sessions. However, the proposed approach and the creation of the new dataset, MEDICA, open avenues for future research and the development of impactful tools for telehealth.

## Introduction

### Overview

The World Health Organization defines mental health as a “state of well-being” that allows a person to lead a fulfilling and productive life and contribute to society [1]. In addition, the World Health Organization estimates that one-fourth of the adult population is affected by a mental disorder [2]. However, there are only approximately 9 psychiatrists per 100,000 people in higher-income countries and only approximately 0.1 psychiatrists for every 1,000,000 people in lower-income countries [3,4]. As stress and pressure continue to increase, leading to poor mental health outcomes, the need for improved tele–mental health care has become critical. Tele–mental health care is the process of providing psychotherapy remotely, typically using Health Insurance Portability and Accountability Act (HIPAA)–compliant videoconferencing [5]. It offers an effective means of accessing mental health services and treatment, transcending geographical and cultural boundaries worldwide, and helps address the chronic shortage of psychotherapists. These services not only remove practical barriers to care, such as transportation, but also offer affordability and direct access to qualified therapists. Therefore, there has been an upward trend in the demand for such services [6]. Despite these undeniable benefits, this emerging treatment modality raises new challenges in patient engagement compared to in-person care. By “engagement,” we refer to the connection between a therapist and patient, characterized by a sense of basic trust and a willingness or interest to collaborate. This connection is essential for the therapeutic process and fosters the development of a strong therapeutic relationship.

### Background

Patient engagement is a critical priority in behavioral health care as it involves establishing effective rapport between health care providers and patients. In the context of mental health, patient engagement is an indicator of a successful therapy session. This multidimensional concept [7,8] encompasses the interaction of cognitive and emotion-related components of the patient’s psychological state. However, one of the challenges in fully understanding and evaluating patient engagement lies in the difficulty of establishing a standardized measurement approach. Patient engagement is a complex and dynamic construct that defies a one-size-fits-all measurement. The diverse factors influencing patient engagement, such as individual characteristics, and health care contexts make it challenging to develop a universally accepted standard of measuring its effectiveness. Traditional quantitative metrics (appointment attendance rates, treatment adherence, and patient satisfaction) often fall short in capturing the depth and richness of patient engagement. While they can provide numerical data, they may overlook the subjective experiences of the interaction and do not necessarily reflect health care provider-patient alliance in treatment. In contrast, qualitative methods such as patient narratives and feedback provide valuable insights but lack scalability, objectivity, and uniformity across different health systems. Therefore, to address these limitations, machine learning techniques are being explored to complement and enhance these traditional methods by leveraging computational power, scalability, and ability to identify patterns, relationships, and insights that may not be otherwise apparent. Some prior works in engagement detection have focused on using a single modality, such as facial expressions [9,10], speech [11], body posture [12], gaze direction [13], or head pose [14], to detect engagement. Combining different modalities has been observed to improve engagement detection accuracy [15-17]. The authors expanded the work of Stanford PBL Lab’s eRing [18] by including information streams such as facial expressions, voice, and other biometric data. Monkaresi et al [19] proposed an approach to detect engagement levels in students during a writing task by not only making use of facial features but also features obtained from remote video-based detection of heart rate. The dataset used was generated by the authors, and they used self-reports instead of external annotation for classification purposes. Chang et al [20] made use of facial expressions as well as body posture for detecting engagement in learners. Fedotov et al [21] proposed the use of audio, facial, and body pose features to detect engagement and disengagement for an in-the-wild dataset that had been created using videos obtained from uncontrolled environments or situations. The distribution of class labels in this dataset is not balanced.

Despite the existence of a variety of such algorithms to perform engagement detection, the results obtained from these approaches (particularly single modality–based approaches) could be misleading in a telehealth setting due to factors such as camera position and resistant or guarded clients. In telehealth appointments, therapists have limited visual data available to them, as they can only view the patient’s face rather than their full body. Asking the patient to adjust their position to gain a better view of their body during telehealth appointments is a potential solution for therapists. However, this approach may lead to communication difficulties, as it can impact the patient’s proximity to the microphone, potentially affecting the quality of audio communication between the patient and the therapist. Therefore, therapists must rely on verbal communication strategies to engage patients than in-person care because they cannot use typical nonverbal cues to convey interest and be responsive to the patient (eg, a handshake at the beginning of a session, adjusting the distance between the patient and health care provider by moving a chair closer or further away, and observing a patient’s response to questions while maintaining eye contact). It is also more difficult for therapists to convey attentiveness because eye contact requires the therapist to look at a camera rather than observe or look at a person. They may also have limited training on telehealth patient engagement, leading to a lack of guidance on measuring patient engagement. Therefore, this highlights the need for a system that can provide feedback on patient engagement by just using the data that are easily accessible, namely, text, audio, and face visuals. Engagement is critical for both retention of patients in care as well as effectiveness of mental health treatment. Developing such a system will make it possible to enhance the quality of tele–mental health and ultimately improve patient outcomes.

### Objective

Taking all this into consideration, we propose and explore a machine learning–based approach that takes visual, audio, and text data as input and estimates the engagement level of a patient with mental health problems through a regression-based approach. We demonstrate the effectiveness of our method in tele–mental health sessions and provide a new dataset called Multimodal Engagement Detection in Clinical Analysis (MEDICA) to advance mental health research by understanding patient engagement levels during therapy sessions. MEDICA consists of 1229 short video clips from mock mental health therapy sessions used by medical schools in their psychiatry teaching curriculum. To the best of our knowledge, MEDICA is the first multimodal dataset that focuses on mental health research and consists of annotations useful for understanding the psychological state of patients with mental health problems during a therapy session.

## Methods

### Datasets

#### MEDICA Dataset

##### Overview

Engagement is an overloaded term, and the definition varies with the application, making it difficult and expensive to collect, annotate, and analyze such data. As a result, we find too few multimodal-based engagement detection datasets currently available for us to use. Our problem statement revolves specifically around detecting patient engagement during a tele–mental health session. In such a setting, the only information we can work with includes the patient’s face and speech (audio and text). There exist datasets such as Carnegie Mellon University Multimodal Corpus of Sentimental Intensity (CMU-MOSI) [22], Carnegie Mellon University Multimodal Opinion Sentiment and Emotion Intensity (CMU-MOSEI) [23], and Stanford Emotional Narratives Dataset (SEND) [24] that capture such settings. However, they are not specifically for engagement detection. Given the lack of a dataset that allows researchers to use multimodal features (video, text, and audio) for engagement, we propose MEDICA, a novel dataset developed specifically to cater to engagement detection using tele–mental health session videos. To use these data to address a broader range of issues related to mental health, we also include labels pertaining to stress and emotions.

To the best of our knowledge, this dataset is one of the first publicly available datasets specifically catering to multimodal research in patient engagement in mental health. Table 1 presents a comparison between MEDICA and other related datasets. Despite the rise in telehealth services and the poor patient-to-therapist ratios for individuals with mental health problems, there are no datasets that even try modeling telehealth sessions to give the community an opportunity to innovate and develop new technologies. MEDICA is a humble attempt by us to kick-start interesting research opportunities. The MEDICA dataset is unique in that it contains multimodal data from multiple sensors including speech and video. These data have been annotated with the engagement levels exhibited by each participant at different points in time.

**Table 1:** Comparison of the Multimodal Engagement Detection in Clinical Analysis (MEDICA) dataset with other related datasets^a^.

Dataset name	Samples	Unique speakers	Modes	Emotion labels	Engagement information	Other mental health cues
RECOLA^b^ [25]	3400	27	{v,a}	Yes	No	Physiological (electrocardiogram and electrothermal activity)
CMU MOSEAS^c^ [26]	715	Multiple	{v,a,t}	Yes	No	—^d^
CMU-MOSI^e^ [22]	2199	Multiple	{v,a,t}	Yes	No	—
CMU-MOSEI^f^ [23]	3228	1000	{v,a,t}	Yes	No	—
SEND^g^ [24]	193	49	{v,a,t}	Yes	No	—
DAiSEE^h^ [27]	9068	112	{v}	No	Yes	—
HBCU^i^ [9]	120	34	{v}	No	Yes	—
In the wild [28]	195	78	{v}	No	Yes	—
SDMATH [29]	20	20	{v,a}	Yes	Yes	—
MEDICA	1229^b^	13	{v,a,t}	Yes	Yes	Hesitation, stress, and attentiveness

^a^Modes indicate the subset of modalities present from visual (v), audio (a), and text (t).

^b^RECOLA: Remote Collaborative and Affective.

^c^CMU MOSEAS: Carnegie Mellon University Multimodal Opinion Sentiment, Emotions and Attributes.

^d^Not applicable.

^e^CMU-MOSI: Carnegie Mellon University Multimodal Corpus of Sentiment Intensity.

^f^CMU-MOSEI: Carnegie Mellon University Multimodal Opinion Sentiment and Emotion Intensity.

^g^SEND: Stanford Emotional Narratives Dataset.

^h^DAiSEE: Dataset for Affective States in E-Environments.

^i^HBCU: historically black college and university.

^j^Current status of the dataset. The size of the dataset will be increased.

##### Acquisition

**Figure 1:**
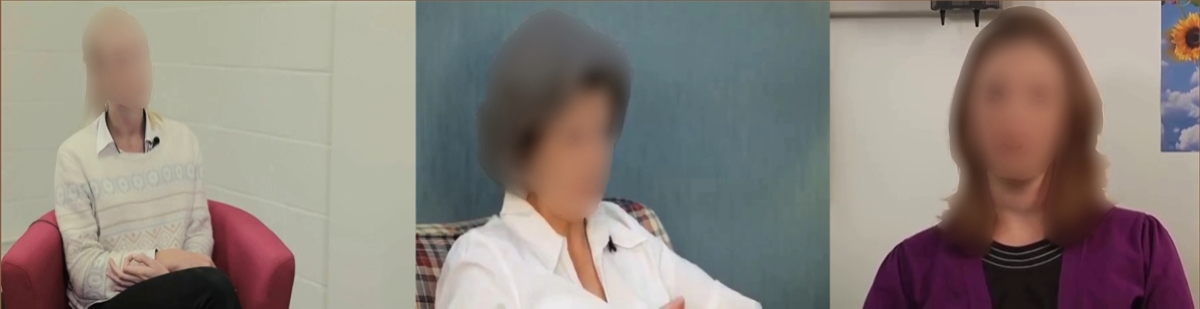
Examples from the Multimodal Engagement Detection in Clinical Analysis (MEDICA) dataset created for mental health research. This dataset has been created using publicly available videos that are usually used for training purposes by different medical schools. The faces of the individuals have been blurred to protect their identity.

MEDICA has been developed by collecting publicly available mock therapy session videos created by different psychiatry medical schools for training their students. The patients in these videos are being advised for depression, social anxiety, and posttraumatic stress disorder. We collected 13 videos, each having a duration of approximately 20 to 30 minutes. We limit the videos to the setup where both the therapist and the patient are not visible together in the same frame. In addition, we take only those videos where there is only 1 patient. Each video has a unique English-speaking patient. The therapists in these videos are actual therapists, while the patients are trained actors who have been dictated to act and react in a particular way. Figure 1 shows the snapshots taken from various video clips in the MEDICA dataset.

##### Processing and Annotation

Our primary focus was to create a dataset that captures the behavior of patients with mental health problems during their sessions. Therefore, we selected only portions of the videos where only the patient was visible in the frames, which appeared at various intervals and durations throughout the recordings. We took these scattered clips and divided them into smaller clips of 3 seconds each, resulting in a dataset of size 1229. We use Moviepy and speech-recognition libraries to extract audio and text from the video clips, respectively. Each video was annotated for attentiveness, stress, and engagement, which were scored on a Likert scale ranging from –3 to 3; hesitation was a binary target variable (yes or no). Humans tend to have multiple emotions with varying intensities while expressing their thoughts and feelings. Therefore, the videos have been labeled for 8 emotions related to mental health: happy, sad, irritated, neutral, anxious, embarrassed, scared, and surprised. This will enable us to develop systems capable of understanding the various interacting emotions of the users.

#### Real-World Data

The videos collected to develop MEDICA were created by the psychiatry schools in controlled settings. By controlled, we mean that the therapists in these videos were real, while the patients were trained method actors who had been instructed in a very detailed manner regarding what to expect and how they should react. While these videos were approved by a team of psychotherapists as being inclusive of potential scenarios and patient reactions, the work would still be meaningless if not tested in the wild, that is, in settings where we have real patients who have not been instructed what to do or how to behave during the session with their real therapist. To accomplish this, we collaborated with 8 child psychotherapists. These psychotherapists are individuals who were trained and licensed as psychotherapists for both children and adults. The administrative staff identified patients that were scheduled for sessions with any of the participating therapists and provided the caregiver contact information. Our research assistant contacted eligible caregivers and provided information related to the purpose, potential risks and benefits, and responsibilities of study participation. They were informed of the study protocol that involved recording 1 to 2 telemental sessions to test our proposed approach. A total of 20 caregivers provided consent to participate in the study. They were provided with instructions on the setup of the equipment that we provided to ensure a clean recording. “Clean” refers to a recording executed with a camera of good quality with appropriate lighting conditions. The equipment included a smartphone with a good quality camera, a ring light with a stand to ensure that the session was recorded in a well-lit environment, and a hot spot internet connection to ensure that the session occurred smoothly without any network glitches. They were also given the assurance regarding preserving the confidentiality of the data being collected. All video recordings were labeled only by study number, with all identifiable information removed and stored in a double password–protected database. Participants were also informed that any facial images other than the caregiver who participated in the session would be deidentified (using methods such as blurring, etc). These steps were taken to ensure that only the caregiver’s level of engagement was measured and analyzed. In addition, this step protected the confidentiality of nonparticipants who did not provide consent.

On average, each of these sessions lasted approximately 20 minutes. The demographics of the caregivers who participated in our real-world experiments can be found in Multimedia Appendix 1. The entire data collection process can be divided into three parts:

1. Before each tele–mental health session of a caregiver with their therapist, a research assistant helped the caregiver with setting up the equipment to record their session. The assistant also ensured that the caregivers were comfortable using the equipment.
2. During the session, we made sure that the tele–mental health session proceeded in the same manner as a typical session. The caregivers were requested to use the equipment provided if possible, but there were no restrictions imposed by us on either the therapist or the caregiver regarding how the session should be conducted. After the presession process, the research assistant would log off. Therefore, during the session, it would be just the therapist and the caregiver having a conversation. No one else from the study would be a part of it. The only thing different about this session was that the caregiver was being recorded using the smartphone given to them. We did not record the therapist.
3. After the session, after the session was complete, a research assistant guided the participant regarding the steps to stop the recording and save the data collected.

**Figure 2:**
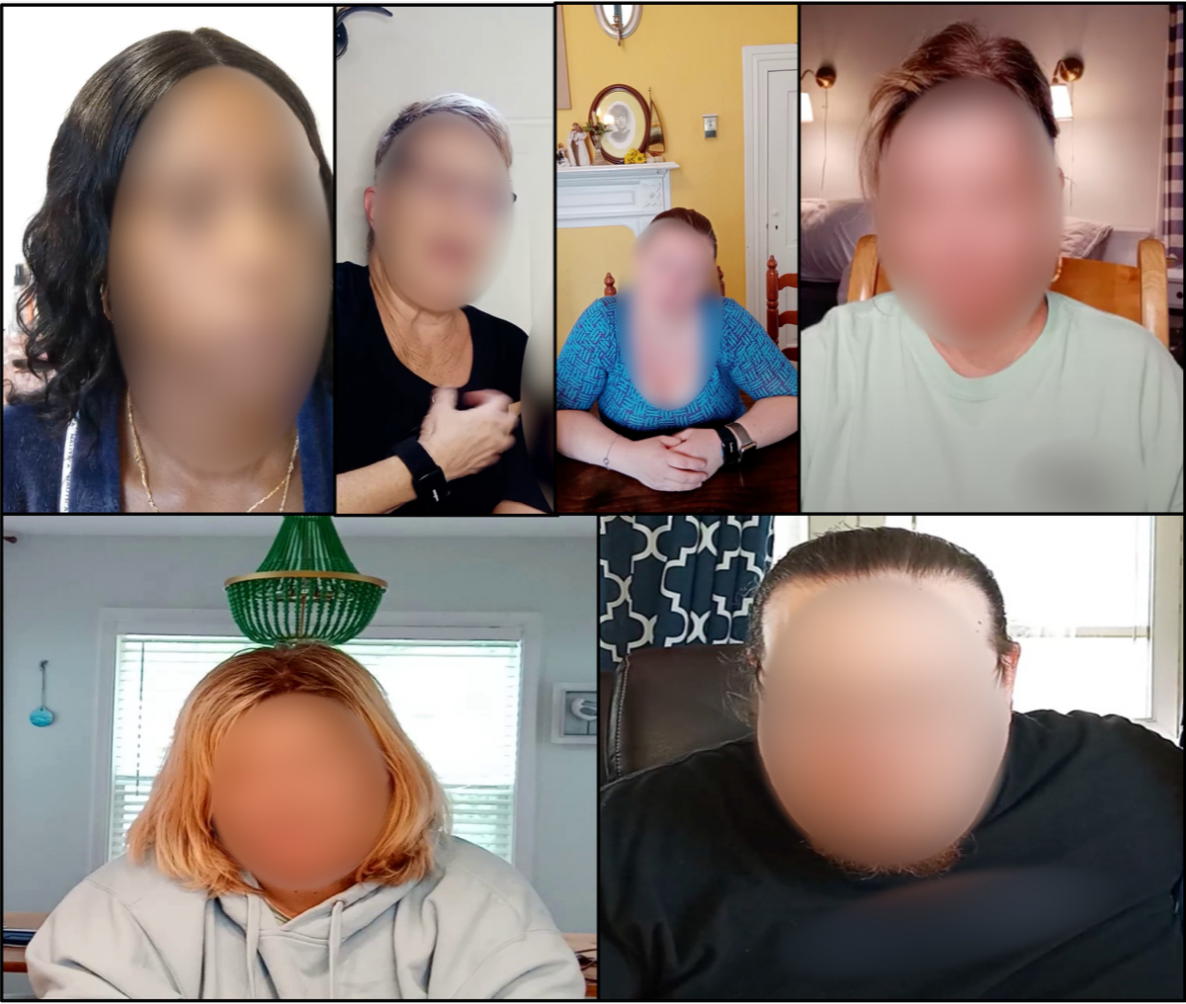
A few frames from the real-world videos we collected. The faces have been blurred here to protect the identity of the patients. However, the consent of the patients was taken to use their unblurred faces as input to our model.

Upon completion of the session, the therapist scored the quality of the collaborative relationship (therapeutic alliance) established between them during the session with the Working Alliance Inventory (WAI) [30,31]. It is used for both child and adult therapy and is a widely studied therapeutic alliance measure. The WAI was modeled on the theoretical work conducted by Bordin [32]. It captures 3 dimensions of the alliance: bond, task, and goals. Extensive tests showed 12 items per dimension to be the minimum length for effective representations of the inventory. A composite score is computed based on these 12 items for each of the sessions conducted. Henceforth, we refer to this score as the WAI score. The WAI scores can range from 12 to 84 [33,34]. Figure 2 shows frames from a few of the video recordings collected.

While efforts were taken to ensure clean video recordings, we still could not use 7 out of the 20 videos collected for any type of analysis. The reasons for it ranged from the video being extremely shaky due to the continuous movement of some participants during their session to not having the permissions to process and analyze videos where the child spoke for the entire session instead of the caregiver. The remaining videos were processed in the same way as MEDICA. At the end of data processing, we obtained approximately 438 clips, each lasting 3 seconds. Unlike MEDICA where we had annotations for every 3 seconds, we had only a single WAI score for the entire session. Therefore, all the 3-second clips obtained from a session were annotated with the WAI score obtained for the session. Any analysis conducted on these data treated all the 438 clips independently.

### Proposed Model Design

**Figure 3:**
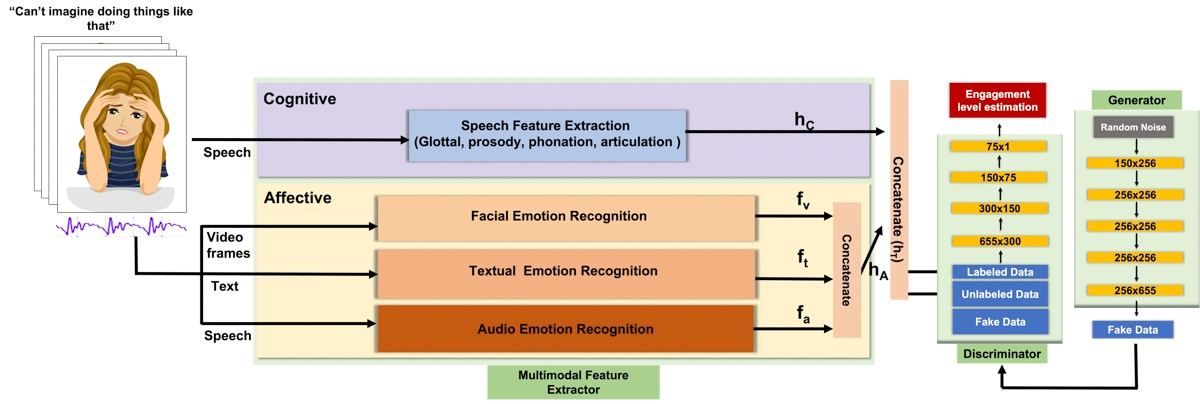
Overall block diagram of the proposed architecture. The features obtained from the 2 modalities, cognitive and affective, are combined to form hT (real) of shape 655×1. The generator takes in random noise (Gaussian) and tries to generate a fake feature map that looks similar to hfakeT that looks similar to hT (real). The discriminator tries to distinguish between hT and hfakeT. The yellow bars in the discriminator and generator refer to linear layers with activation function as leaky rectified linear activation unit. The shape of the linear layers used has been written on the bars. The number of linear layers used can be changed depending upon the dataset size and task at hand.

Figure 3 presents an overview of our engagement estimation model. It has 2 key components: *multimodal feature extractor* and *semisupervised learning network*. We discuss each of them in detail in the subsequent sections.

#### Multimodal Feature Extractor

##### Overview

Multimodal feature extractor in general refers to a system or algorithm that extracts and combines features from multiple modalities, such as audio, video, text, and other types of data, to provide a more comprehensive representation of input data. The goal of multimodal feature extraction is to capture complementary information from different sources, which can lead to better performance in various tasks. In our model, the multimodal feature extractor is used to comprehend the psychological state of the patient with mental health problems. We take inspiration from literature in psychology and psychiatry to build an extractor that can enable the learning network to recognize, understand, and evaluate engagement as close as possible to that of a psychotherapist. Therefore, we proposed a multicomponent approach to build the feature extractor and introduce 2 modules, namely, cognitive and affective states, to capture the different cues used by psychotherapists to assess their patients. As mentioned earlier, the visual data available are limited. Therefore, apart from the visuals, we also need to make use of verbal information, that is, understanding the speaking style of the person (audio) and what the person said (text).

Therefore, given this information corresponding to a participant, the multimodal feature extractor outputs a feature vector *h_T_*. *h_T_* is obtained after concatenating the feature vectors *h_C_* and *h_A_* obtained from the cognitive and affective state modalities (components of the multimodal feature extractor), respectively. We now discuss the cognitive and affective state modalities in more detail in the subsequent sections.

##### Cognitive State Modality

The cognitive state involves comprehending complex concepts and issues and acquiring difficult skills. It conveys deep (rather than surface level) processing of information, where the person gains a critical or higher-order understanding of the subject matter and solves challenging problems.

Psychotherapists usually measure and evaluate the cognitive state of the person using the mental status examination, typically conducted via in-person interviews or self-evaluations, to gauge memory, thinking, and the extent of understanding of the topic of discussion. There has been a lot of work around determining biomarkers for detecting signs of a person’s cognitive state. However, these methods are either offline or fail to consider various essential perceptual indicators. Recently, there has been a lot of work around using speech as a potential biomarker for detecting cognitive decline. For instance, stress negatively affects the cognitive functions of a person, and this can be easily detected using speech signals. Moreover, speech-based methods are attractive because they are nonintrusive, inexpensive, and can be done in real time. Four distinct features have proven to be highly effective in identifying signs of cognitive impairment and are increasingly utilized to detect conditions like Alzheimer’s and Parkinson’s diseases. These features include glottal (*f_g_*), prosody (*f_pr_*), phonation (*f_ph_*), and articulation measures (*f_ar_*), each offering unique insights into speech characteristics associated with cognitive decline.

Glottal features are acoustic measures used to characterize speech under stress. The glottal is the space between the vocal cords in the larynx, and the opening and closing of the vocal cords create sound during speech. During periods of stress, there is an aberration in the amount of tension applied in the opening (abduction) and closing (adduction) of the vocal cords. Prosody features characterize the speaker’s intonation and speaking styles. Under this feature, we analyze variables such as timing, pitch, and loudness during the production of speech. Phonation refers to the production of vocal sounds by the vibration of the vocal cords. In individuals with cognitive decline, phonation is often characterized by bowing and inadequate closure of the vocal cords. These changes can lead to instability and irregularity in the vibration of the vocal cords, which in turn affects the production of speech. They are analyzed in terms of features related to perturbation measures, such as jitter (temporal perturbations of the fundamental frequency), shimmer (temporal perturbation of the amplitude of the signal), amplitude perturbation quotient, and pitch perturbation quotient. Apart from these, the degree of unvoiced is also included. Articulation features refer to the movements of the lips, tongue, and jaw during speech production. Reduced amplitude and velocity of these movements can impact speech intelligibility. The analysis of articulation is primarily based on the first 2 vocal formants F1 and F2, which correspond to the frequency of the sound waves produced by the vocal tract.

For a given audio input, we extract these features using *librosa* [35] and *praat* [36] libraries. Therefore, we define features corresponding to cognitive state as the concatenation of these 4 audio features. Therefore, cognitive state features

h_c_ = concat (f_g_, f_pr_, f_ph_, f_ar_) **(1)**

##### Affective State Modality

The affective state encompasses emotional reactions such as excitement, boredom, curiosity, and anger. The range of affective expressions will vary based on individual demographic factors (eg, age), cultural backgrounds or norms, and mental health symptoms.

To understand the affective state, we check if there exists any inconsistency between the emotions perceived and the statement the person made. Balomenos et al [37] and Porter and ten Brinke [38] suggest that when different modalities are modeled and projected onto a common space, they should point to similar affective cues; otherwise, the incongruity suggests distraction, deception, etc. In other words, if E1, E2, and E3 represent the emotions perceived individually from what the patient said (text), the way they said it or sounded (audio), and how they looked or expressed (visuals), respectively, then the patient would be considered engaged if E1, E2, and E3 are similar; otherwise, they would be considered disengaged. Therefore, we adopt pretrained emotion recognition models to extract affective features corresponding to audio, visuals, and text from each video sample separately.

Audio features (*f_a_*) are extracted using mel-frequency cepstral coefficients (MFCC) from audio clips available in the dataset. MFCC is a commonly used audio feature extraction technique in signal processing and machine learning. It is based on the concept of the mel scale, which is a nonlinear transformation of frequency that is more perceptually relevant to human hearing. By applying MFCC to audio signals, we can extract features that capture important aspects of the sound, such as pitch, timbre, and spectral shape. These features are often used in speech and music analysis as well as in various machine learning applications, including emotion recognition and speaker identification. In our work, the extracted MFCC features from audio are then passed as input to a multilayer perceptron network trained for emotion recognition in speech, specifically using the data available in the Crowd-source Emotional Multimodal Actors Dataset (CREMA-D) to extract affective features from the audio data. Through this method, we obtained a feature vector f_a_, for each audio clip, which provides valuable insights into the emotional characteristics of the speech.

Visual features (*f_v_*) correspond to the affective features extracted from video frames to understand the emotions expressed through facial cues of the patient. We used a deep learning architecture called Visual Geometry Group-B (VCG-B), which has been pretrained as described in the study by Arriaga et al [39], to extract the affective features. We modified the output dimensions of the second last layer of the network to produce a feature vector *f_v_* of length 100.

Text (*f_t_*), characterizing the emotions, sentiment, and other affective cues present in the text, is obtained using a Bidirectional Encoder Representations from Transformers(BERT)–based model, which has been pretrained on the GoEmotions dataset [40]. The Bidirectional Encoder Representations from Transformers model works by processing the text input through a multilayer neural network that is trained to predict the context of each word based on its surrounding words. This allows the model to capture the complex relationships between words and their meanings, which are essential for understanding the affective context of the text.

Therefore, we represent the affective state of the patient as a concatenation of *f_a_*, *f_v_*, and *f_t_*. Hence, affective state features

h_A_= concat (f_a_, f_v_, f_t_) **(2)**

Therefore, the overall output of the multimodal feature extractor is a feature vector *h_T_* *which c*an be defined as

h_T_ = concat (h_C_, h_A_)

The learning network discussed in the next section makes use of this.

### Learning Network

In this section, we discuss the second part of our proposed model, that is, the semisupervised learning network. Different machine learning techniques can be explored to solve the task of predicting the level of engagement. However, obtaining a large amount of high-quality labeled data to train a robust model for predicting patient engagement is inevitably laborious and requires expert medical knowledge. Considering that unlabeled data are relatively easy to collect, we propose a semisupervised learning–based solution. Semisupervised learning enables us to deploy machine learning systems in real-life applications (eg, image search [41], speech analysis [42,43], and natural language processing) where we have few labeled data samples and a lot of unlabeled data. Some prior works have also explored semisupervised learning for engagement detection in nonmedical domains. One of the earliest studies in this area is by Alyuz et al [44], where they consider the development of an engagement detection system, more specifically emotional or affective engagement of students, in a semisupervised fashion to personalize systems such as intelligent tutoring systems according to their needs. Nezami et al [45] conducted experiments to detect user engagement using a facial feature–based semisupervised model. Most state-of-the-art semisupervised learning methods use generative adversarial nets (GANs) [46].

GANs are a class of machine learning models and typically have 2 neural networks competing with each other to generate more accurate predictions. These 2 neural networks are referred to as the generator and the discriminator. The generator’s goal is to artificially manufacture outputs (ie, fake data) that could easily be mistaken as real data. The goal of the discriminator is to identify the real from the artificially generated data. In trying to generate high-quality outputs, the generator learns to capture the different possible variations in the input variables, and therefore, the data manifold well. This is extremely helpful when we may not be able to access data containing a wide variety of similar engagement-related cues visible across different patients. In general, the training process of GANs involves a dynamic interplay and competition between the generator and discriminator networks. Both the generator and discriminator networks improve their abilities iteratively. The generator aims to generate more realistic samples, while the discriminator aims to become better at distinguishing between real and generated (artificial) data. The eventual goal of the training process is for the generator and discriminator to reach Nash equilibrium [47]. Once the equilibrium is achieved, no player (generator or discriminator) has any incentive to deviate toward a more optimal position, that is, no player has a better strategy given the choices of the other player.

Taking all this into consideration, we propose using a multimodal semisupervised GAN as our learning network for regressing on the levels of patient engagement. This type of machine learning model combines multiple modalities (in this case, cognitive and affective modalities) to generate realistic samples (or features) that can fool the discriminator. The term *semisupervised* refers to the network’s ability to use a limited amount of labeled data alongside a larger amount of unlabeled data to enhance its accuracy. Such a network will generalize better and be more robust compared to previously defined semisupervised learning approaches. It is different than the semisupervised GAN framework SR-GAN proposed by Olmschenk et al [48] in 2 main ways. First, our network is multimodal, whereas SR-GAN is not. Second, unlike the SR-GAN generator, which focuses on generating realistic images, our GAN’s generator is designed to create realistic feature maps that mimic the multimodal feature vector *h_T_*, derived from the fusion of cognitive and affective state modalities (as discussed in the previous section). The generator takes Gaussian noise as input and produces a synthetic feature vector *h_fakeT_*, aiming to deceive the discriminator into classifying it as *h_fakeT_*. The discriminator’s role, in turn, is to distinguish *h_T_* as a fake representation and correctly identify *h_T_* as the genuine feature vector.

Overall, our multimodal semisupervised GAN can therefore be divided into 3 parts: multimodal feature extractor, generator, and discriminator.

For the purpose of training, we make use of the following four standard loss functions, namely, labeled loss (*L_lab_*), unlabeled loss (*L_un_*), fake loss (*L_fake_*), and generator loss (*L_gen_*):

1. *L_lab_*—this loss is similar to typical supervised learning regression losses, where the goal is to assess the network’s ability to estimate the engagement level accurately, as close as possible to the ground truth (ie, the actual engagement level annotated for the data sample). Therefore, we compute the mean squared error of model output (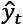, ie, predicted level of engagement) with ground truth (*y_t_*, ie, actual level of engagement).



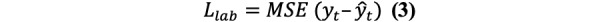



2. *L_un_*—in semisupervised GANs, the labeled dataset contains a small portion of data with labels, while most of the data are unlabeled. The goal of the L_un_ loss function is to improve the performance of the generator by minimizing the distance between the feature spaces of the labeled and unlabeled datasets. This helps to ensure that the generator produces feature maps that are consistent with both labeled and unlabeled data, thereby improving the generalization performance of the model. By minimizing the distance between the feature spaces of labeled and unlabeled datasets, the model can effectively learn from both labeled and unlabeled data, which is particularly useful in cases where labeled data is scarce.



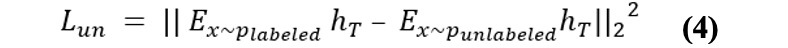



3. *L_fake_*—it is a loss function used to update the discriminator network. The objective of this loss is to maximize the distance between the feature space of unlabeled dataset and fake features generated by the generator. This enables the discriminator to distinguish the real and fake data features better.



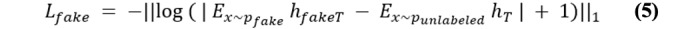



4. *L_gen_*—the generator network’s objective is to produce feature vectors *h_T_* (fake) that can be passed off as real feature vectors *h_T_*: L_gen_ updates the generator network to do just that. L_gen_ encourages the generator to learn the underlying distribution of unlabeled data features (real) and generate fake features that match the feature statistics of the real features.



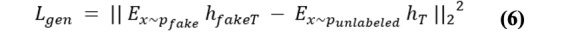



Similar to SR-GAN, we also make use of a gradient penalty (P) to keep the gradient of the discriminator in check, which helps convergence. The gradient penalty is calculated with respect to a randomly chosen point on the convex manifold connecting the unlabeled feature vector *h_T_* to the fake feature vector *h_T_*. We compute it as



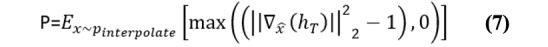



Here, *p_interpolate_* examples are generated by *αp_unlabeled_ + (1 – α)p_fake_* for *α ~ U (0,1)*.

*U (0,1)* represents a uniform distribution over the range from 0 to 1.

The overall loss function used for training the network is:

L = L_lab_ + L_un_ + L_fake_ + L_gen_ + λP **(8)**

### Overall Pipeline

In this section, we present an overview of how the various components of our proposed approach interact to estimate the engagement level of a patient with mental health problems during a tele–mental health session. We provide insights into the working mechanism of our model and shed light on the underlying processes involved in engagement level estimation in a multimodal setting.

As depicted in Figure 3, the proposed model involves data preprocessing, which includes extracting video frames, audio signal, and text data from the input video clip. To capture engagement dynamics over small time scales, we analyze engagement at the microlevel, considering video clips of a few seconds duration. This aligns with the person-oriented analysis suggested by Sinatra et al [49]. Specifically, for the MEDICA dataset and real-world data, we consider video clips of 3 seconds duration. All samples, labeled and unlabeled, from the dataset are treated as *real* data for the semisupervised GAN. The multimodal feature extractor (consisting of the cognitive and affective state modalities) is used to obtain the *h_T_* features from the preprocessed visual, audio, and text data. *h_T_* is considered real. In parallel, the generator network creates *h_fakeT_* (ie, fake *h_T_*) features using gaussian noise as input. The labeled and unlabeled *h_T_* features obtained from the multimodal feature extractor and the *h_fakeT_* features obtained from the generator are then input to the discriminator network. In addition to distinguishing between real (*h_T_*) and fake (*h_fakeT_*) features, the discriminator network outputs the engagement level of the patient with mental health problems as a continuous value.

The subsequent sections outline the different studies being conducted to verify our approach.

### Study 1: Testing Our Proposed Approach on MEDICA

The purpose of the first study is to test the applicability of our approach to estimate the levels of engagement exhibited by the participants present in the videos. The test was conducted on the MEDICA dataset. In addition, we compared the performance of the proposed model against other similar frameworks to understand its effectiveness. To test the models, we partitioned the dataset in a ratio of 70:10:20 for training, testing, and validation. Motivated by recent works in clinical psychotherapy [50-52], we used the standard evaluation metric of root mean square error (RMSE) to evaluate our approach. Smaller the RMSE value, better is the performance of the model for the engagement estimation task.

### Study 2: Ablation Studies

The proposed model design involves the introduction of 2 modalities, namely, affective and cognitive. In this study, we explored the contribution of these 2 modalities for the purpose of engagement detection. We aimed to test the effectiveness of the model with and without these modalities. Therefore, we ran the overall pipeline described earlier by removing either one of the modalities corresponding to affective and cognitive states and reported our findings. The tests were conducted on the MEDICA dataset. Similar to our previous study, we performed the evaluation using RMSE.

### Study 3: Analysis on Real-World Data

Evaluating the model only on MEDICA is not sufficient to prove the usability of the model in the real-world setting. Therefore, this study aimed to test our approach on data collected from real-world scenarios. The study involved testing the proposed model that has been trained on MEDICA using real-world data. We also compared the performance of our proposed model against other baselines explored in study 1 on the real-world data. Apart from testing our model’s usability for the real world, we also tested its capability to perform on unseen real-world data, which is different from the data (MEDICA) it has been trained on. Contrary to previous experiments where we compared the performance on engagement estimation values, in this test, we will study the correlation of the model-estimated engagement values with the WAI score. This will help us to also test whether the scores estimated by our model are clinically useful for therapists.

### Ethical Considerations

Both the MEDICA dataset and the real-world data collection processes have been approved by the institutional review boards (#HP-00092966) at both the University of Maryland College Park’s computer science department and University of Maryland Baltimore medical school.

Participation of the caregivers while collecting the real-world data was completely voluntary. They could withdraw anytime without any negative consequences. They were informed about the purpose of the study and the analysis that would be conducted using the data collected.

The therapist and the participating caregiver were given the option of allowing us to store videotaped recordings for development of future studies or to request to have all videotape sessions destroyed after a 2-year period. The video recordings were stored in our laboratory databases and subject to strict University of Maryland privacy protocol, including encryption and password protection. While the research is ongoing, only select project personnel with internal clearance can have access to the data. In addition, we ensured that the data are untampered using standard cryptographic hash functions. During the video storage process, we deidentified any facial images beside the health care provider or caregiver who are recorded (eg, sibling interrupts session) so that they are not recognizable on the recorded video. All data related to enrollment, demographics, and questionnaires will be stored in an electronic database at the University of Maryland and will be double password protected with only select research members having access. The participating caregiver was given an individual ID number, and their data were deidentified to ensure it could not be linked back to them.

While the participating caregiver may not directly benefit from the study, they received 1-month free internet hot spot and a Fitbit, which were provided as part of the real-world study equipment. They were compensated with a US $20 gift card after returning the equipment.

## Results

The semisupervised models were trained on an NVIDIA GeForce GTX 1080 Ti GPU with a batch size of 512 and a learning rate of 0.0001 for 50 epochs.

### Study 1: Testing Our Proposed Approach on MEDICA

The purpose of the first study is to demonstrate the ability of our model to estimate the level of engagement exhibited by the patient in the video. This study was performed on the MEDICA dataset. As our proposed methodology leverages a semisupervised approach, we extracted labeled samples from MEDICA and unlabeled samples from the CMU-MOSEI dataset. After preprocessing, we extracted 12,854 unlabeled data points from CMU-MOSEI. We split the 1229 labeled data points from MEDICA into 70:10:20 for training, validation, and testing, respectively. Therefore, the split of the labeled training data to unlabeled training data points was 860:12,854. In addition, the ground truth engagement levels corresponding to the training data points in the MEDICA dataset were normalized to fall within a range of 0 and 1. We compared our model with the following state-of-the-art methods for engagement detection:

1. Kaur et al [28] used a deep multiple instance learning–based framework for detecting engagement in students. They extracted local binary pattern on three orthogonal planes (LBP-TOP) features from the facial video segments and performed linear regression using a deep neural network to estimate the engagement scores.

2. Nezami et al [45] performed a semisupervised engagement detection using a semisupervised support vector machine.

In addition to being state-of-the-art, these methods can be used in a telehealth setting like ours. We used the publicly available implementation proposed by Kaur et al [28] and trained the entire model on MEDICA. Nezami et al [45] do not have a publicly available implementation. We reproduced the method to the best of our understanding.

Table 2 summarizes the RMSE values obtained for methods described by Kaur et al [28] and Nezami et al [45], including ours. We observed an improvement of at least 40%. Our approach is one of the first methods of engagement estimation built on the principles of psychotherapy. The modules used, specifically cognitive and affective states, help the overall model to effectively mimic the ways a psychotherapist perceives the patient’s level of engagement. Similar to psychotherapists, these modules also look for specific engagement-related cues exhibited by the patient in the video.

**Table 2:** Comparison of RMSE values obtained from various methods for estimating levels of engagement on the Multimodal Engagement Detection in Clinical Analysis dataset.

Method	RMSE^a^ for engagement
Kaur et al [28]	0.96
Nezami et al [45]	0.17
Our approach	0.10

^a^RMSE: root mean square error.

### Study 2: Ablation Studies

To show the importance of the different components (affective and cognitive) used in our approach, we run our method on MEDICA by removing either one of the modules corresponding to affective or cognitive states and report our findings. Table 3 summarizes the results obtained from the ablation experiments. We observe that the ablated model (ie, only using affective [A] or cognitive [C] modules) does not perform as well as the model that includes both modules. To understand and verify the contribution of these modules further, we leveraged the other labels (stress, hesitation, and attention) available in MEDICA and performed regression tasks using our proposed architecture on all of them. We observed that mode C performed better when predicting stress and hesitation values. Mode A performed better in estimating a patient’s level of attentiveness. These results agree with our understanding of cognitive state and affective state. Therefore, the combination of affective and cognitive state modes helps in efficiently predicting the engagement level of the patient.

**Table 3:** Ablation experiments on the Multimodal Engagement Detection in Clinical Analysis dataset. We ran our proposed model using only 1 modality at a time (either affective or cognitive) and compared the performance with that of using both modalities together.

Modality	RMSE^a^ for engagement	RMSE for stress	RMSE for hesitation	RMSE for attention
Affective	0.24	0.15	0.146	0.07
Cognitive	0.3	0.13	0.16	0.08
Affective + cognitive (our approach)	0.10	0.12	0.14	0.1

^a^RMSE: root mean square error.

### Study 3: Analysis on Real-World Data

**Figure 4:**
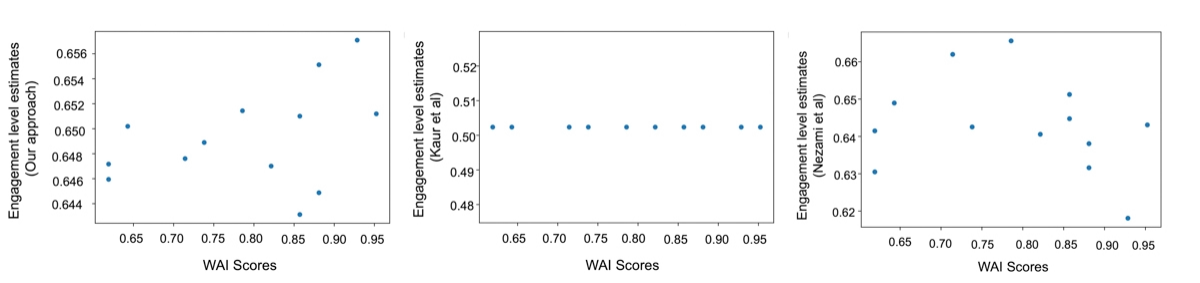
Scatterplot between the engagement level estimations obtained from each method for each session: (A) the plot between engagement level estimations obtained from our approach and the Working Alliance Inventory (WAI) scores, (B) the plot between the engagement level estimations obtained by Kaur et al [28] for each session and the WAI scores, (C) the plot between the engagement level estimations for each session obtained by Nezami et al [45] and the WAI scores.

Our model that has been trained for estimating engagement levels using the MEDICA dataset was tested on the processed real-world data. WAI scoring is based on certain observations the therapist makes during the session with the patient. The score obtained from our model was different from the WAI score, but we claim that similar to WAI, our estimates also captured the engagement levels of the patient well. If this is indeed the case, then both WAI and our estimates should be correlated. As discussed earlier, a single WAI score is reported by the therapist (health care provider) for the entire session. Unlike WAI, which reports a single score per session, our method, along with those proposed by Kaur et al [28] and Nezami et al [45], performs microanalysis, generating engagement estimates at multiple time points within a session. To enable a fair comparison, we calculated the mean of the engagement estimates across each session for all methods. The mean engagement estimates across sessions were as follows: Kaur et al [28] had a mean of 0.50 (SD: 7.82e-17), Nezami et al [45] had a mean of 0.64 (SD: 0.017), and our approach had a mean of 0.65 (SD: 0.006). We subsequently examined each of their correlations with the corresponding WAI scores across sessions. Given that WAI scores and engagement estimates are measured on different scales, WAI scores were normalized to a 0-1 range for consistency with the engagement values from the methods. We found that Kaur et al [28] obtained a correlation score of –0.03, Nezami et al [45] obtained a score of –0.24 and our approach obtained a much better score of 0.38. We plot the engagement level estimations obtained for each session against its corresponding WAI score, as shown in Figure 4. The plot corresponding to our approach shows a noticeably better alignment with the WAI score trends compared to other methods [28,45].

Additionally, instead of just taking the mean, we also took the median of the engagement level estimates available at different instances of the sessions. The average median of the engagement estimates across sessions for different methods were as follows: Kaur et al [28] had an average median of 0.50 (IQR 0.0), Nezami et al [45] had an average median of 0.64 (IQR 0.02), and our approach had an average median of 0.65 (IQR 0.007).

We then examined the median of the engagement estimates for each session, obtained using different methods, and assessed their correlation with the WAI scores. In this case, Kaur et al [28] obtained a correlation score of 0, Nezami et al [45] obtained a score of –0.18 and our approach obtained a relatively high score of 0.40. These findings reinforce that our model captures WAI patterns more effectively than prior methods. Its stronger positive correlation with the WAI scores indicates its capability to better estimate engagement levels during therapy sessions.

The conceptual model of our proposed approach is also supported by the theoretical work by Bordin [32]. According to this theory, the therapist-provider alliance is driven by 3 factors: bond, agreement on goals, and agreement on tasks; these factors align well with the features identified in this work. While bond would correspond with the affective state, goals and task agreement correspond with the cognitive state. The merit of the approach by Bordin [32] is that it has been used for child and adult therapy, and it is one of the more widely studied therapeutic alliance measures. Therefore, it is no surprise that our approach can work well to provide an estimate of engagement levels in a tele–mental health session.

## Discussion

### Principal Findings

In this study, we developed a machine learning–based model that integrates features related to cognitive and affective psychological states to predict patient engagement levels during tele–mental health sessions. The input to our proposed algorithm is the visual, audio, and text data available, while the output is the engagement level of the patient. Our investigation demonstrated the significant promise of our proposed method, achieving an average improvement of 40% in RMSE for predicting engagement levels on the newly introduced MEDICA dataset compared to prior works. In real-world settings, our method provided patient engagement estimates that closely aligned with the trends observed in WAI scores assigned by therapists based on their sessions [30,31]. This study highlights the potential of our multimodal engagement estimation algorithm to enhance the quality of telehealth services and ultimately improve patient outcomes.

### Comparison With Prior Work

Patient engagement is recognized as a pivotal determinant of a successful therapy session, particularly in the realm of mental health care [53]. Current psychiatric literature [54-56] has extensively examined a range of strategies and methodologies designed to foster [57] and sustain patient engagement during therapy sessions. These strategies are crucial for achieving optimal therapeutic outcomes. There has been a growing interest in applying artificial intelligence (AI) to mental health [58-62], with innovative approaches being explored to enhance patient care. However, despite the proliferation of AI-based methods in mental health applications, there remains a noticeable gap in research specifically focused on understanding and quantifying engagement. This gap is even more evident in the context of tele–mental health [63]. The transition from traditional in-person therapy to digital platforms has introduced unique challenges, as conventional indicators of engagement, such as body language [64], eye contact [65], and physical presence, maybe less discernible or entirely absent in web-based settings.

Our research addresses this critical gap by pioneering an approach that estimates patient engagement specifically in the tele–mental health context. This work is the first of its kind to target engagement estimation in this setting, offering a novel perspective that has largely been overlooked in previous studies. While the broader concept of engagement has been explored by AI in various domains, existing methods [28,45,66] have not been adapted or tested within the unique demands of mental health care, particularly in a remote or web-based environment. From an algorithmic standpoint, previous works have explored both unimodal and multimodal learning-based networks to understand engagement. Unimodal approaches focus on individual modalities, such as speech, body posture, biometric data (eg, heart rate variability and skin conductance) [67,68], gaze direction [69], and head pose [70]. Multimodal approaches [71] integrate various signals, leveraging the complementary strengths of each modality to provide a more holistic understanding of engagement. In contrast to these works, our research focuses on using the types of information that are readily available during a tele–mental health session, specifically audio, video, and text data. This approach is designed to be practical and easily implementable in real-world telehealth settings. This also ensures that the engagement estimation process is minimally disruptive to the therapeutic experience during the telehealth session. We validated our approach using our newly introduced dataset, MEDICA. In addition, we tested it on data collected from real-world therapy sessions. Our method achieved state-of-the-art performance in both cases.

### Strengths and Implications

In this work, we explored the multidimensional and temporally dynamic nature of patient engagement [72]. Our method estimates engagement at regular short intervals. These characteristics of our approach align perfectly with the person-oriented analysis discussed by Sinatra et al [49]. We released a new dataset, MEDICA, to enhance mental health research, specifically toward understanding the engagement levels of patients attending therapy sessions. To the best of our knowledge, there is no other multimodal dataset that caters specifically to the needs of the mental health–based research. In addition, while there are some image-based [9,27,28] or sensor information–based datasets, there is no dataset that addresses the possibility of exploring engagement detection using visual, audio, and text modalities. We verified the usefulness of our machine learning method using 3 studies. Study 1 demonstrates the ability of our model to estimate the engagement level exhibited by the patient in video. It was done to understand the usefulness of the proposed model in comparison to other existing engagement estimation methods such as those by Nezami et al [45] and Kaur et al [28]. Our approach reported an RMSE [50-52] of 0.1, which on an average is 40% less than the RMSE reported by other methods (Table 1). Our study is one of the first to estimate engagement specifically for patients with mental health problems in a tele–mental health setting. The modalities used, specifically cognitive and affective states, propel the overall algorithm to mimic the way a psychotherapist perceives the patient’s level of engagement. As part of study 2, we were interested to understand the contribution of the 2 modalities (cognitive and affective states). The experiments conducted as part of this study are also termed as *ablation* as it involves running the network pipeline using only 1 of the 2 modalities available at a time. We leveraged the annotations in MEDICA on stress, hesitation, and attention to dig deeper regarding the contribution of the cognitive and affective state modalities. We ran the same ablation experiments on not only patient engagement but also stress, hesitation, and attention estimation. While the combination of affective and cognitive states gives better results (RMSE of 0.1), it was interesting to note that the observations obtained supported the theoretical discussion about the 2 states. Cognitive state modality was able to understand stress (RMSE of 0.13) better. Affective state that was built on the concept of capturing distraction using emotion inconsistency between visual, audio, and text data helped relate better with the understanding of attention (RMSE of 0.07). These results agree with our understanding of cognitive state and affective state discussed earlier.

WAI is a popular measure of patient engagement used by many mental health care providers. Therefore, in study 3, we wished to understand and check if the values being estimated by our method related well with the trends observed in WAI. We did so by computing the correlation scores between WAI and the values estimated by our proposed model. In addition, to test the effectiveness of our approach, we also compared the correlation scores obtained between WAI and patient engagement values estimated by other existing methods. Positive values are preferred because they indicate similarity in distribution behavior. Negative values would indicate that the trends observed in WAI and the engagement values predicted are different and opposite. The results (Table 2) revealed that our model was able to capture the trends in WAI much better than other engagement estimation methods, reinforcing its clinical relevance. The correlation strength observed between WAI and our method’s estimated engagement values is better than other approaches, but it also indicates the need for further investigation. The trend is positive, suggesting that we have successfully modeled engagement to some extent. Further analysis will provide opportunities to enhance and strengthen the relationship between WAI and the engagement values estimated by machine learning.

### Limitations and Future Work

One of the primary limitations of the proposed algorithm is that engagement predictions may not be optimal in case of occlusions or missing modalities, data corruption due to low internet bandwidth, and integration of wearable devices with our model. We recognize that this situation is likely to occur in a tele–mental health session and plan to incorporate solutions for it in our future work. In addition, we aim to explore ways to make the predictions more explainable, allowing psychotherapists to receive evidence-based suggestions to support their final decisions.

### Conclusions

Telehealth behavioral services, delivered via videoconferencing systems, have emerged as a cost-effective, dependable, and secure option for mental health treatment, particularly in recent times. However, gauging patient engagement, a critical mental health care standard, remains challenging in telehealth due to the absence of nonverbal cues and sensory data such as heart rate. To address this, we propose a novel multimodal semisupervised GAN that leverages affective and cognitive features from psychology to estimate engagement levels using visuals, audio, and text from video calls. This approach can significantly enhance social interactions and assist psychotherapists during tele–mental health sessions. Engagement is typically assessed through patient reports, which are susceptible to response bias, and current measures such as “show rate” and “patient satisfaction” do not accurately reflect the health care provider–patient alliance. Our model demonstrates its effectiveness on both our newly introduced dataset called MEDICA as well as real-world data. Given the lack of systematic engagement measurement and the limited training for health care providers on telehealth engagement, our proposed system offers a promising solution to these challenges, promoting better patient-health care provider interactions and making telehealth more effective.

## Data Availability

The datasets generated during and analyzed during this study are available from the corresponding author on reasonable request.

## Appendix

Multimedia Appendix 1 Demographic information on the real-world experiment participants.

## Abbreviations

AI: artificial intelligence
CMU-MOSEI: Carnegie Mellon University Multimodal Opinion Sentiment and Emotion Intensity
CMU-MOSI: Carnegie Mellon University Multimodal Corpus of Sentimental Intensity
GAN: generative adversarial net
HIPAA: Health Insurance Portability and Accountability Act
MEDICA: Multimodal Engagement Detection in Clinical Analysis
MFCC: mel-frequency cepstral coefficients
RMSE: root mean square error
WAI: Working Alliance Inventory
CREMA-D: Crowd-source Emotional Multimodal Actors Dataset
VGG-B: Visual Geometry Group-B
BERT: Bidirectional Encoder Representations from Transformers
LBP-TOP: local binary pattern on three orthogonal planes
