# Supplementary material for: Developing a Machine Learning–Based Automated Patient Engagement Estimator for Telehealth: Algorithm Development and Validation Study
Source: JMIR Form Res. 2025 Jan 20;9:e46390. doi: 10.2196/46390 (PMC11791444; doi:10.2196/46390)
Supplement: Multimedia Appendix 1 [file formative_v9i1e46390_app1.docx]

Table S1: Demographic information for 19 caregivers participating in the experiment. One participant didn’t share demographic details.

| Measure |  | Count | Percentage |
| --- | --- | --- | --- |
| **Gender** |  |  |  |
|  | Male | 1 | 5% |
|  | Female | 18 | 95% |
| **Race** |  |  |  |
|  | American Indian | 0 | 0% |
|  | Black American | 6 | 31% |
|  | Caucasian | 12 | 63% |
|  | Native Hawaiian | 1 | 5% |
|  | Biracial | 0 | 0% |
| **Ethnicity** |  |  |  |
|  | Non-Hispanic | 19 | 100% |
|  | Hispanic | 0 | 0% |
| **Household Income** |  |  |  |
|  | < $20000 | 2 | 10% |
|  | $20000-$40000 | 6 | 31% |
|  | $40000-$60000 | 2 | 10% |
|  | $60000-$80000 | 0 | 0% |
|  | $80000-$100000 | 2 | 10% |
|  | >$100000 | 5 | 26% |
